# Exploring the provision and support of care for long-term conditions in dementia: A qualitative study combining interviews and document analysis

**DOI:** 10.1177/14713012231161854

**Published:** 2023-03-07

**Authors:** Jessica Rees, Alexandra Burton, Kate Walters, Claudia Cooper

**Affiliations:** Division of Psychiatry, 4919 University College London, London, UK; Department of Behavioural Science and Health 4919 , University College London, London, UK; Primary Care and Population Health, 4919 University College London, London, UK; Centre for Psychiatry and Mental health, Wolfson Institute of Population Health, 4617 Queen Mary University of London, London, UK

**Keywords:** dementia, long-term conditions, chronic conditions, healthcare, management, qualitative, COVID-19

## Abstract

**Background:**

The challenge of managing multiple long-term conditions is a prevalent issue for people with dementia and those who support their care. The presence of dementia complicates healthcare delivery and the development of personalised care plans, as health systems and clinical guidelines are often designed around single condition services.

**Objective:**

This study aimed to explore how care for long-term conditions is provided and supported for people with dementia in the community.

**Methods:**

In a qualitative, case study design, consecutive telephone or video-call interviews were conducted with people with dementia, their family carers and healthcare providers over a four-month period. Participant accounts were triangulated with documentary analysis of primary care medical records and event-based diaries kept by participants with dementia. Thematic analysis was used to develop across-group themes.

**Findings:**

Six main themes were identified from eight case studies: 1) Balancing support and independence, 2) Implementing and adapting advice for dementia contexts, 3) Prioritising physical, cognitive and mental health needs, 4) Competing and entwined needs and priorities, 5) Curating supportive professional networks, 6) Family carer support and coping.

**Discussion:**

These findings reflect the dynamic nature of dementia care which requires the adaptation of support in response to changing need. We witnessed the daily realities for families of implementing care recommendations in the community, which were often adapted for the contexts of family carers’ priorities for care of the person living with dementia and what they were able to provide. Realistic self-management plans which are deliverable in practice must consider the intersection of physical, cognitive and mental health needs and priorities, and family carers needs and resources.

## Background and objectives

As people age, the risk of developing long-term conditions increases (Barnett et al., 2012). So too, does the risk of developing dementia (Corrada et al., 2010). The number of people living with dementia worldwide is predicted to double every twenty years (Dementia Forcasting Collaborators, 2022; Prince et al., 2015). Long-term conditions are common in people with dementia (Poblador-Plou et al., 2014), with an estimated 8 in every 10 people with dementia living with another long-term condition, such as diabetes, hypertension and stroke (Public Health England, 2019). With a healthcare system that is often designed around single condition services, improving the treatment and management of multiple long-term conditions is an important challenge for health services (Coulter et al., 2013).

When living with a chronic disease, self-management is a daily task (Lorig & Holman, 2003). This task is severely impacted by the symptoms of dementia (Ibrahim et al., 2017). Self-management support from family carers, social care and primary care can facilitate the optimal management of long-term conditions to prevent hospitalisation, slow cognitive decline, and enable people with dementia to live independently at home for longer (Bordier et al., 2014; Doraiswamy et al., 2002; Fox et al., 2014). A recent systematic review highlighted the importance of collaboration between family members, healthcare professionals and homecare workers to support long-term condition management for people with dementia (Rees, Tuijt, et al., 2020).

The presence of dementia complicates healthcare delivery and the development of person-centred physical health care plans (Bunn, Burn, et al., 2017). The ideal of person-centred care lies at the centre of many models of care (Coulter et al., 2013; Wagner et al., 1996) and health and social care policies (Department of Health, 2016; NHS, 2014; NHS England, 2019) which have been developed to manage complex care needs. Such care is proposed to be holistic, integrated and organised by need and not disease (World Health Organisation, 2015). A greater understanding of the needs of people with dementia and multiple long-term conditions is required to ensure condition-specific guidelines are relevant (Scrutton & Brancati, 2016). In a recent secondary analysis of qualitative interviews, we highlighted the importance of identifying changes in self-management ability as dementia progresses to adapt care accordingly (Rees, Burton, et al., 2020). This study provided a breadth of information regarding what stakeholders consider priorities for care delivery, however few studies have explored in-depth the experiences of people with dementia and long-term conditions. The triangulation of multiple data sources can also enable a greater understanding of health and social issues (Farmer et al., 2006).

In the present study, we used multiple data sources to qualitatively explore how care for long-term conditions in dementia was provided and supported in the community. As our study took place in 2020/1, we also explored how care provision for long-term conditions in dementia were affected during the pandemic.

## Methods

### Setting and sample

Participants were eligible to take part if they had a documented diagnosis of dementia of any severity, and an additional diagnosed long-term condition. This was defined as a health condition requiring ongoing support from primary care or significant elements of self-management. We used prevalence studies to identify common long-term conditions in people with dementia (Browne et al., 2017; Public Health England, 2019). List of eligible conditions included, diabetes, asthma, Chronic Obstructive Pulmonary Disease (COPD), arthritis, stroke, and heart failure/disease. We included participants with dementia living in the community, including those who lacked capacity to consent to research. Abiding by the Mental Health Capacity Act of England and Wales (2005), the lead author assessed capacity of people with dementia. If the person with dementia lacked capacity to decide whether to take part, family carers were invited to act as personal consultee. People with dementia who had capacity were not required to have a family carer who provided regular support to participate in the study.

We included family carers providing regular support (at least weekly contact) for the person with dementia to manage their health-related activities. This included medication and appointment management and broader aspects of health such as exercise and nutrition. Health and social care professionals were deemed eligible if they were identified by participants with dementia and/or their family carers as supporting management of or providing care for long-term conditions. We used purposively sampling to ensure a diverse range of experience including type of long-term condition, stage of dementia, age, gender, ethnicity of the person living with dementia and extent of involvement of family members and health or social care professionals. We recruited participants via social media, previous dementia research studies, Join Dementia Research and six general practices supported by the National Institute for Health and Care Research, Clinical Research Network (North Thames) using letters of invitation or by direct approach by healthcare professionals. Health and social care professionals identified by people with dementia (and family carers) taking part in the study were invited to participate via email. Written informed consent was obtained from all participants.

### Data collection

This study involved two complementary remote data collection methods. Firstly, JR undertook qualitative semi-structured interviews based on a topic guide (see Supplemental Material), followed by a series of participant led interviews over 4 months with people living with dementia and family carers via telephone or video call. Topic guides focused on the person with dementia’s health history, daily long-term condition management and support, understanding of current care plans, and impact of COVID-19 on condition management. Subsequent interviews explored how issues raised at the initial interview evolved, and any new issues which arose or where managed. Interviews were audio-recorded and transcribed verbatim. JR took observational field notes of dyadic interactions between participants with dementia and those involved in their care during video interviews.

Secondly, we undertook document analysis of consultation notes and care plans provided by primary care. We requested, with participant consent, the last ten consultation notes and care plans from general practitioner (GP) practices of participants. All identifiable information was redacted prior to being stored electronically on university systems. JR also invited participants with dementia if they were able, to complete event-based diaries. Over a two-week period, participants recorded specific events related to their management of long-term conditions, what they did to look after their health and who was involved.

### Data analysis

Using a reflexive thematic analysis approach (Braun & Clarke, 2019) from a post-positivist, or critical realist perspective (Bhaskar, 1979) we analysed data using NVivo 12 (QSR International Pty, 2018). Based on approaches which focus on the uniqueness of individual experience (Smith et al., 2009) we used a dual approach to analysis by developing themes within case studies before considering themes across cases. Following analysis of each case, co-authors (JR, AB, KW, CC, CC, KW, AB) met to discuss key concepts and reflected on interpretation of meaning and emerging themes. To triangulate data across sources, stakeholders and time, we used a ‘back-and-forth’ approach (Bowen, 2009). For example applying codes from the analysis of interview transcripts to the content of documents and vice versa. We then grouped themes within each case based on shared patterns of meaning and used discussion to develop and refine across group themes. To present themes, we used a thematic structure drawing on examples of individual experiences from case studies to further evidence central organising concepts (Clarke & Waring, 2018).

### Reflexivity

Reflexivity is core component for qualitative research (Rankl et al., 2021). During data collection and analysis, the lead author acknowledged their positionality as an ‘outside researcher’(Hellawell, 2007) with no experience of dementia or chronic illness. JR kept a reflective journal of the influence of her personal and academic biography on the research process, in addition to professional biographies of co-authors including an old age psychiatrist, GP and qualitative health researcher.

## Findings

### Sample characteristics

Data were collected from 18 participants (that formed eight case studies, one case included two people living with dementia) between September 2020 and May 2021. Participants comprised of nine people with dementia, seven family carers and two healthcare professionals (GP and neurologist). Of the seven family carer participants, four were female and three were male. Four were spousal-carers and three child-carers. Table 1 provides the demographic characteristics of participants with dementia.

**Table 1.** Characteristics of participants with dementia.

	Number of participants
**Age**	
70–79	3
80–89	3
90+	3
**Gender**		
Female	5	
Male	4	
**Ethnicity**		
White	7	
South Asian	2	
**Marital status**		
Married	6	
Widowed	2	
Divorced	1	
**Living arrangements**		
With family	8	
Alone	1	
**Type of dementia**		
Alzheimer’s disease	5	
Vascular	1	
Mixed	1	
Frontotemporal	1	
Posterior cortical atrophy	1	
**Stage of dementia**		
Early	2	
Moderate	4	
Late	3	
**Long-term conditions**		
Diabetes	3	
Cardiovascular conditions*	6	
Visual impairment	2	
Hearing impairment	2	
Respiratory conditions	3	
Renal failure	2	
Arthritis	3	
Depression/anxiety	3	

*included hypertension, stroke and heart failure.

JR conducted 26 interviews (between one and five per case study). Primary care records were collected for all nine participants with dementia. Two people with dementia kept an event-based diary. Observational field notes were recorded from four video interviews. The majority of interviews were conducted on a one-to-one basis with a family carer. For three case studies, dyadic interviews were conducted with the person with dementia and their family carer. Three people with dementia had capacity to consent to research, of whom two participated in interviews independently. For the remaining six, a family member acted as a personal consultee.

### Qualitative analysis findings

Across eight in-depth, diverse case studies (see Table 2) we identified six over-arching themes regarding how care for long-term conditions is provided and supported in dementia. 1) Balancing support and independence focused on the tension between the value of independence in health management for people with dementia, and the need for increased support by family carers as cognition declined. 2) Implementing and adapting advice for dementia contexts related to how family carers adapted care to overcome symptoms of dementia and in the absence of a holistic approach to care from services, family carers attempted to integrate advice for dementia and other long-term conditions. 3) Prioritising physical, cognitive and mental health needs considered the inevitable trade-offs between caring for physical, cognitive and mental health, often creating a hierarchy, which shifted according to changing needs. 4) Competing and entwined needs and priorities described the interconnectedness of the needs of people with dementia and their family carers and the influence of this on proxy-decision making. 5) Curating supportive professional networks focused on the role of the family carer in curating supportive professional relationships, from primary, secondary and social care, to facilitate healthcare. 6) Family carer support and coping considered decisions about when to access support and how this was influenced by perceptions of capacity to cope with the demands of caring. This included decisions to involve homecare workers to support the management of long-term condition in dementia. In the theme descriptions below, each name is a pseudonym, matched to gender and ethnicity of participants with dementia.

**Table 2.** Overview of participant case studies.

Case studies	Case description	Data collection
1. Edith and Scott	Edith is in her 90s and has mixed Alzheimer’s disease and vascular dementia. She lives alone, supported by her son Scott and homecare workers who visit four times a day. Main issues are reduced mobility due to knee osteoarthritis and low mood. She uses a walking frame and is restricted by risk of falls to the first floor as she cannot use stairs. She accesses services (vaccination, opticians) via home visits.	Dyadic video interview (x1) Family carer video interview (x4) Consultation notes (x1)
2. Doris and Bert	Doris is in her mid-70s and has severe Alzheimer’s disease. Main concerns are nutrition, frailty and toileting. Husband Bert manages her diet, medication and appointments. Doris had chronic Myeloid Leukaemia for 16 years and retains a close relationship with the oncologist and pharmacist from these times.	Family carer telephone interview (x1) Consultation notes (x1)
3. Harold and Dora	Harold is in his mid-80s and has early Alzheimer’s disease. He has insight into his declining cognition and increasing dependence on his wife, Dora. His main issues are visual impairment (glaucoma, cataracts) for which he takes eye drops. He has atrial fibrillation and is under investigation for heart failure due to progressing swollen legs. He self-monitors his heart rhythm via an app and jogs daily to manage low mood.	Person with dementia telephone interview (x3) Family carer telephone interview (x2) Event-based diary (x1) Consultation notes (x1)
4. Fiona and Declan	Fiona is in her early 70s and has posterior cortical atrophy, causing severe dementia. Issues include swallowing, continence, agitation and poor sleep. Husband Declan manages her medication including a daily inhaler for COPD. Declan is recovering from open heart surgery during data collection, so daughter, Maeve, who has been furloughed due to the COVID-19 pandemic, supports with housework, food shopping and medication collection.	Family carer telephone interview (x4) Consultation notes (x1)
5. Margaret, Jonathan and Sophie	Margaret and Jonathan are husband and wife who both have dementia. Margaret, in her 80s has Alzheimer’s disease, anxiety and depression, COPD and renal failure. Jonathan, in his 90s, has vascular dementia since a stroke and diet-controlled diabetes. They are supported by their daughter Sophie who is their live in carer paid through personal budgets.	Dyadic video interview (x1) Family carer video interview (x1) Family carer telephone interview (x1) Healthcare professional interview (x1) Consultation notes (x1)
6. Hassan	Hassan is in his late 80s and has mild Alzheimer’s disease. His main issues are insulin-controlled diabetes, arthritis and he has a colostomy. He lives in a multi-generational household with two sons and his daughter in law.	Person with dementia telephone interview (x2) Consultation notes (x1)
7. Samira and Sarah	Samira is in her 70s and has primary progressive aphasia (fluent variant). Her main issue is arthritic knee pain and a trapped nerve in her back, for which she has decided to have an elective operation. She manages her own medication for asthma and diabetes, supported by her daughter Sarah, who lives with her in a multi-generational household.	Dyadic video interview (x1) Dyadic telephone interview (x3) Event-based diary (x1) Consultation notes (x1) Healthcare professional interview (x1)
8. Albert and Jean	Albert is in his 90s and has moderate Alzheimer’s disease. He uses a walking frame to reduce his risk of falls as his balance worsened following a stroke. He has hearing loss. Albert lives with his wife Jean and is supported by a homecare worker who visits daily to facilitate personal care.	Dyadic video interview (x2) Family carer telephone interview (x3) Consultation notes (x1)

NB. Each name is a pseudonym, matched to gender and ethnicity of participants with dementia.

### Theme 1. Balancing support and independence

Balancing the need for increased support with declining cognition was an important process due to the value of independence for people living with dementia. For Hassan, a participant living with mild to moderate dementia, it was important to feel he did not depend on others. He stated ‘*I do it myself’* eleven times across both interviews relating to his self-administration of insulin twice daily. GP consultation notes record that he was ‘*taking all his meds.’* However, it is unclear whether this was the GP’s own assessment or Hassan’s self-report.

This strong sense of independence may have been related to insight into increasing dependence on others. Harold, another participant in the early stages of dementia, only had partial insight into his self-management abilities and his need for an increasing level of support from his wife, Dora, to manage his medication, eye drops and to advocate on his behalf in appointments. In his event-based diary, Harold stated he self-managed his medication: “*I have my medication (many pills) well organised.*” Dora was conscious of a need to balance Harold’s care needs and his need to perceive himself as autonomous, as she did not ‘*want him to lose his independence*.’

In the context of dementia, the desire for independence could at times lead to compromised quality of disease management. In the quote below, Hassan recounts support from his family when experiencing hypoglycaemic faints, suggesting he may have had difficulties self-managing his insulin injections.

*“Once in the bathroom I passed out and couldn’t get up. Couldn’t get up at all. So I had to call my son and he had to call the ambulance and all that. And I was lying down in the bathroom for some time. And then slowly they pulled me up. This happened two, three times.” (Hassan, Interview 1)*

A commonality across these accounts was the difficult balancing act for family carers, to ensure appropriate management of long-term conditions. Dora describes reaching a tipping point in this balance, where she intervened to ensure Harold received help for progressive cardiac failure.

*“Now he has got a very swollen feet, extremely swollen and he just dismissed it as if nothing was wrong with it. I want him to go to the doctor. I have to insist for him to make an appointment, and at this stage I don’t want him to lose his independence. I just try to help as much as I can but I want to go to the doctor with him for that reason otherwise he does everything for himself.” (Dora, Interview 1)*

In other scenarios, family carers felt able to accept decisions they did not agree with. Samira was successfully able to take an active role in managing her healthcare and care decisions with daily support from her daughter, Sarah. This included making many decisions independently, for example about having surgery.

*“I didn’t really want her to have it. I thought obviously age, diabetes and the health and stuff, it’s not really conducive to do it. But I mean she did it. It was okay and the pain came back. And there was always the possibility the pain would not go away…But it’s part of, the complications were she probably end up with more back pain and she was happy to go through with the surgery.” (Sarah, Interview 2)*

A facilitator of this appeared to be the slow rate of Samira’s cognitive decline enabling her to maintain relative independence. In her event-based diary, Samira showed an awareness of her medical issues: *“I have to see the doctor to discuss about my leg pains to discuss about root injection or surgery or an epidural.”* For diabetes management, Sarah provided support to monitor Samira’s blood glucose levels.

### Theme 2. Implementing and adapting advice for dementia contexts

Implementing the advice given by healthcare professionals in the home to manage long-term conditions was a challenge for family carers, who adapted care to overcome symptoms of dementia. Bert and Declan, both husband carers for a person in the later stages of dementia, described needing to adapt condition-specific advice to dementia contexts. Both described the challenge of interpreting symptoms when their wives were unable to verbally communicatee with them due to dementia. Bert described how his ability to interpret symptoms with limited feedback was facilitated by his knowledge of his wife, Doris.

*“You rely on the patient you say what works, how are you feeling, did that hurt, if I push you here what does… it is notoriously difficult because you get no, all medication requires patient feedback. And with Alzheimer’s that gradually diminishes to zero. You have to interpolate [sic], and if you know someone very well like [Doris] and I do, that makes life…very easy. It must be incredible difficult for people who handle this with strangers I would guess.” (Bert, Interview 1)*

One common difficulty was around managing swallowing difficulties associated with severe dementia. Bert considered carefully whether Doris’s reluctance to eat related to swallowing difficulties or apathy. Declan described needing to request orodispersable tablets due to Fiona’s swallowing difficulties. Declan perceived a lack of integrated services for dementia and physical health that made caring harder. He felt primary care focused on physical health management and reported how little he perceived dementia to be mentioned. There was a sense in his accounts that integration of medical advice happened at home and was his responsibility.

*“Err…well they are dealing with the attending problems of dementia, but they never really speak about the dementia itself or how I’m coping with it or what’s happening in her life. How she and I are coping with it. They completely ignore that. Just medical problems they’re concerned with. They don’t really want to know about anything else you know. I think that’s probably in the domain of [area] people as well. The memory service and that sort of thing.” (Declan, Interview 4)*

### Theme 3. Prioritising physical, cognitive and mental health needs

The co-occurrence of physical, cognitive and mental health needs in people with dementia led to inevitable trade-offs during the prioritisation of care. For Edith, who lived with moderate dementia, fall risk management strategies meant she was housebound, despite a deleterious impact that her son perceived on her quality of life through reduced social interactions. Edith was prepared to accept such compromises as her priority was remaining at home, but her son felt this contributed to her low mood.

*“I would like to live in my own home for as long as possible even if it means not being able to go down the stairs.” (Edith, Memory service care plan)*

Managing the risk of falls was also a key issue for Albert, who lived with late onset dementia, and his wife Jean. When I first met Albert and Jean, initiation of anti-dementia medication was postponed as side-effects included dizziness, which could ultimately have increased the risk of falls. In later interviews Jean described how Albert’s dementia had worsened. His memory service notes record how he can become ‘*another self’* being ‘*rude*’ and ‘*bad tempered’* when forgetting things. At this point, supported by primary care, Jean re-considered the need for antipsychotic medication ‘*although not been keen on medication in the past*’. She described the difficult trade-off between the need to reduce behaviours that challenged and to avoid medication that increased falls risk.

There were a number of instances across accounts where physical health care appeared to be prioritised, either over cognitive or mental health needs. In terms of improving cognition, there was a sense of futility, while physical conditions were perceived as addressable. Harold described not discussing his dementia with his GP as he ‘*assume[d] it’s just going to progress*.’ In his diary, Harold described his mental health as ‘*not stable’* as he conveyed the impact of his deteriorating cognition on his low mood. This was not reflected in his consultation notes, which focused on his physical health, specifically vision impairment and cardiac problems. Harold’s proactive approach to physical health management appeared not to extend to his management of cognitive and mental health.

*“In a way it’s just there, it’s like having arthritis. You can’t suddenly get rid of it. It goes on for maybe a couple of days and then I’m alright. I’m alright today and it’s very hard to explain it. It’s not something I had before I got the diagnosis. And I think it was a reaction to the diagnosis as much as anything. You know we all, all human beings rely hugely on memory and I thought I wasn’t going to have any memory. Pretty awful. And of course now I forget things which I ought to remember and that reminds me that things aren’t good.” (Harold, Interview 3)*

### Theme 4. Competing and entwined needs and priorities

At times the needs of family carers and the people living with dementia felt competing and entwined. Decisions were often made based on dyadic needs, that family carers usually needed to judge alone. Scott, for example, limited Edith’s contact with services, as he considered some services to be logistically difficult to attend and feared that visits might be ‘*too disruptive’* resulting in a negative impact on her health. For example, the ability to attend appointments due to mobility issues were discussed as a factor in Scott’s decision to remove Edith from the waiting list for her cataract operation.

*“As I said, I felt rotten that I had to, well she’s been taken off the list. Removed. Because other people need the slots. I would have kept it on, I would have kept on but eventually it was not right for me, morally wrong to keep saying yes, yes she’ll be there, she’ll be back there again, she'll be in there again for another. When I know that she couldn't possibly get there.” (Scott, Interview 1)*

In her memory service care plan, Edith felt she could ‘*make it down the stairs if she’s careful’* suggesting how her perception on risk management may differ from Scott’s. Similarly, for Margaret and Jonathan, a married couple with dementia, their GP considered that while health management was enabled by their daughter Sophie who loved and cared for them, it was also complicated by her anxieties. Sophie described fearing that her caring responsibilities might overwhelm her, but also that her parents would not be able to cope with knowing they had dementia.

*“And the anxieties that are projected from the carers are very valid, because they are with them the whole time, but you wonder how much you are treating the carer rather than actually treating your patient.” (GP, Interview 1)*

*“When I go to the hospital I say please don’t mention my parents condition [dementia]. I know personally it would worry them, they wouldn’t be able to handle it. I mean obviously they were told in the beginning, they’ve forgot. But doctors have said no it’s important they should know. But I know my parents would…and if someone told me and I couldn’t remember that I had it I would panic. It would make me worse.” (Sophie, Interview 1)*

Albert and Jean both needed support from homecare workers, and it was often practical to consider their needs collectively. For example, a physiotherapist attending the home to provide neurotherapy treatment for Jean’s vertigo was able to assess Albert following a fall reported in our second interview.

*“It just so happened that the physiotherapist had come here to see me… later that morning. So she very kindly, because they know [Albert] anyway from previous sessions of physio. So she did all the observations, you know, take his temperature and pulse, blood pressure. And everything was normal so when I reported that to the doctor.” (Jean, Interview 2)*

*“Review by community therapy team following a fall at home...the nature of the fall seemed to be mechanically-related due to losing his footing when transferring to the sofa.” (Letter from Physiotherapist to GP)*

### Theme 5. Curating supportive networks

To navigate the health system and access care when needed, family carers actively curated and managed relationships with professionals. In some cases they developed personal closeness, which at times proved useful to circumvent challenges of access to care during the pandemic. Sarah’s close relationship with the neurologist was perceived to facilitate care for Samira through improved communication.

*“Communication is very easy. In some ways, it should be, and it is mostly for all patients I see with these long-term conditions, they have either my email or some form of point of contact, my secretaries’ email. My phone number. One of the nurse specialists. I mean in [Sarah’s] case it’s very easy isn’t it because you know I’ve got to know her quite well. So yeah she can contact me any time.” (Neurologist, Interview 1)*

Similarly, Bert recounted how the oncology department kept in touch reporting ‘*we don’t want you to fall through the cracks*.’ Curating such supportive networks meant professionals were aware of need and adapted care accordingly. For example, consultants coordinated care to accommodate Bert’s preference of not wishing to attend hospital during COVID-19 due to concerns about Doris’s frailty.

*“Doris needed a blood test check. And I was reluctant to go into hospital for that. So they, the senior nurse there who I’ve got on with for years, very kindly said she’d sort out with the GP to do it…But when I was talking to [name] the neurologist he spotted on the computer notes that the consultant, [name], had been in touch with the GP, and that he’d arranged, they are in the same hospital, so he arranged for one of the girls, well the nurses there, to take Doris’s blood and send it off. So they arranged it that way and I haven’t had to go to doctors at all.” (Bert, Interview 1)*

### Theme 6. Carer support and coping

Family carer attitudes towards acceptance of support influenced their ability to cope, with COVID-19 also impacting the availability of support. Furlough enabled Fiona’s daughter Sophie to provide care while Declan recovered from open-heart surgery. On the other hand, Sophie experienced decreased support from other family members who visited less due to concerns of COVID-19 transmission. This sense of additional responsibility contributed to high levels of carer burden. However, some decision making support from family was retained remotely, for example relating to COVID-19 vaccination.

*“Yeah it is left up to me but I’m really really indecisive. Really indecisive. Like with the vaccination for example I wasn’t sure I was a bit worried about letting them have it, and I asked all my other members of family. I’m not very assertive. And I’ve got good support with my family.” (Sophie, Interview 1)*

Family carer acceptance of support was an important theme across these narratives. Declan organised homecare worker support yet discontinued after one session as it felt difficult to organise and was experienced as unsupportive.

*“No paid carers at all. I’ve been reluctant to do that…If I left her with someone I wouldn’t go out or do anything, I’d only sit out there fretting. So I’m really managing myself.” (Bert, interview 1)*

This reflected a wider sense in his narratives that ‘*nothing can be done*’ to manage Fiona’s challenging behaviour.

*“Oh yes, from several of the people that neurology said they would contact through doctor referrals they have been in touch. But really there’s very little any of them can do… Because they can’t really, [Fiona] just…there’s not much they can do with her you know.” (Declan, Interview 3)*

A similar view was expressed by Sophie who, based on negative past experience, felt reluctant to involve homecare workers, and again by Bert who expressed difficulties in sharing caring responsibilities for his wife. In both accounts family carers also expressed concerns that although the caring burden was overwhelming, sharing it with homecare workers risked increasing their burden, due to the additional coordination of care involved, or through fear that they would be let down if the service was not provided.

## Discussion

### Main findings

We identified six interrelated themes using multiple data sources to explore how care for long-term conditions in dementia is provided and supported. Balancing the need for increased support with declining cognition was an important process due to the value of independence for people with dementia. Family carers were responsible for adapting care to overcome the symptoms of dementia, and actively curated and managed relationships with professionals to access support when required. The co-occurrence of physical, cognitive and mental health needs led to inevitable trade-offs during the prioritisation of care needs. Interconnection between family carers and the people with dementia they supported led to a sense of competing needs and priorities. Finally, the availability of support for family carers was impacted by COVID-19, acceptance of which was influenced by perceptions of their own coping ability.

Loss of independence has been identified as a key stage in the dementia journey (Forbes et al., 2012). From the perspective of participants in the early stages of dementia in this study, the value of independence in health management was an important priority. The transition from self-management to dependence in the context of long-term conditions in dementia has previously been described (Bunn, Burn, et al., 2017). As cognition declined, participants in this study became increasingly dependent on family carers to ensure quality of disease management. Research to date has focused on the transition experiences of family carers providing support for multiple long-term conditions in dementia (Lam et al., 2020; Ploeg, Northwood, et al., 2020). By considering perspectives across case studies, this study highlights the interacting nature of transitions in this context. For example, the lack of insight of a person with dementia into the severity of health issues (heart failure) led to the increased involvement of his wife (primary carer) and a transition to greater dependence on the family carer for care of long-term conditions.

The concept of interdependencies is well recognised in person-centred dementia care (Manthorpe & Samsi, 2016). Family carers in this study were responsible for adapting care for long-term conditions to overcome symptoms associated with dementia. In addition to personal and medical tasks, husband carers of people with dementia and multiple long-term conditions have been found to adopt the role of ‘protector’ of personhood (Sanders & Power, 2009). A particular challenge noted by participants related to the integration of physical and cognitive advice where this was not done by health professionals. Findings from this study emphasise how people with dementia and family carers were adaptive in prioritising needs in a holistic way, which may be physical, mental or cognitive depending on the stage of cognitive or physical decline. Salient issues identified in previous literature include those requiring immediate or ongoing attention such as safety and pain (Ploeg, Garnet, et al., 2020). This reflects our findings, where functional needs such as mobility and reducing the risk of falls took higher priority than mental health in the hierarchy of needs.

Recent theories recognise illness management as a dyadic phenomenon. Lyons and Lee (2018) in their Theory of Dyadic Health Management suggest that addressing incongruence in illness appraisal between patients and their family carers is key to achieving better health through collaborative management. Findings from this study suggest how interconnecting needs and priorities can lead to incongruence between the wishes of the person with dementia, and the decision making of the family carer. Difficulties in separating the perspectives of people with dementia and their family carers have been reported in the pain literature (Amspoker et al., 2021). Another goal of the Theory of Dyadic Health Management relates to optimising the health of both dyad members (Lyons & Lee, 2018). Spousal carers of people with dementia are often also managing their own health conditions (Lam et al., 2020). Participants in this study described relinquishing caregiving responsibility whilst recovering from surgery.

Previous research into the management of diabetes in dementia suggests how regular contact with a supportive professional improves management by providing flexible individualised care (Bunn, Goodman, et al., 2017). In this study, to facilitate the management of long-term conditions, family carers of people with dementia curated support from wider family, healthcare professionals and homecare workers. These findings are consistent with the House of Care Model, where engaged and informed patients interact with healthcare professionals to work in partnership to achieve personalised care (Coulter et al., 2013). For some participants in this study, relationships with professionals lead to improved communication which facilitated access to remote consultations during the pandemic. Changes in service provision during COVID-19 have been found to impact the quality of consultations with primary care particularly when addressing physical health needs (Tuijt et al., 2021). Findings from this study suggest how pandemic-related factors also impacted availability of support, contributing to family carer burden. Participants accounts reflected how such factors impact decision making processes, specifically around vaccination and the involvement of homecare workers. Factors associated with discontinuing homecare during the pandemic include risk of COVID-19 transmission and concerns around adequate use of Personal Protective Equipment (Giebel et al., 2020).

### Strengths and limitations

To our knowledge, this paper presents the first study to use multiple qualitative data sources, such as interviews and document analysis, to explore how care for long-term conditions is provided and supported for people living with dementia at home. Collection of data from multiple sources enables triangulation, or the comparison of different data sources for comprehensive insight, and to improve validity of findings (Mays & Pope, 2000; Roper & Shapira, 2000). Strengths of this study include the diverse range of participant experiences, including type of dementia, long-term condition, level of cognitive impairment, degree of support and interaction with services. We recognise the variability in available data sources per case. To mitigate this, we treated each case as individual during analysis prior to developing themes across groups. In the final write up, we ensured all case studies were used to evidence themes. Recruitment of healthcare professional and homecare workers was limited due to service pressures during COVID-19. Consultation notes were particularly useful during analysis to compare the accounts of people with dementia and/or family carers with those who provided care. The majority of interviews were conducted via telephone which may have been preferred in this population due to familiarity and access (Unnithan, 2021). However, conducting research remotely may have changed the degree of inclusivity for participants with dementia and excluded some participants with low digital literacy or sensory impairment. As data collection for this study spanned from September 2019 to May 2020, the pandemic context meant that findings relate to experiences at an unprecedented time of healthcare delivery.

### Implications

Living with multiple long-term conditions in dementia requires the management and prioritisation of a variety of physical, cognitive and mental health needs. Our findings highlight the dynamic nature of dementia care, which has implications for people with dementia and their family carers accessing services annually for long-term conditions. Previous research suggests that older people do not necessarily differentiate between co-existing conditions, thus find it challenging when services focus on a single disease (Ploeg et al., 2017). To consider needs holistically, care and support should be adapted to the context of dementia at a service level. Our findings illustrate how intertwined psychosocial and physical needs are, yet how psychological needs can be overlooked if they are conceptualised as an inevitable sequelae of dementia. The bi-directional impact of physical, cognitive and mental health needs suggest they should be considered together in clinical practice. These findings accord with the integrated logic model of care which posits that psychosocial, mental, cognitive and physical needs should be addressed simultaneously due to their influence on each other (Hansen et al., 2017).

Due to the integral role of family carers, these findings underline the importance of conceptualising people with dementia and those who support their care as an interdependent team (Lyons & Lee, 2018). Research recommendations by the National Institute for Health and Care Excellence include exploring effective care planning methods for people who do not have regular contact with a carer (NICE, 2018). People who dementia who live alone are more likely to use homecare services, and experience unmet social and medical needs (Miranda-Castillo et al., 2010). Thus, exploring how long-term conditions in people with dementia without support are managed would be an important direction for future research.

### Conclusions

Due to the dynamic nature of dementia care, support for long-term conditions in dementia is required to be adapted in response to changing need. As dementia progresses, it is important that care is organised using a family-centred approach that acknowledges the daily realities of implementing care recommendations in the community. Realistic self-management plans which are deliverable in practice must consider the interacting nature of physical, cognitive and mental health needs, and acknowledge that these needs are often conflated with family carers in the context of dementia.

## Supplemental Material

Supplemental Material - Exploring the provision and support of care for long-term conditions in dementia: A qualitative study combining interviews and document analysis Click here for additional data file. Supplemental Material for Exploring the provision and support of care for long-term conditions in dementia: A qualitative study combining interviews and document analysis by Jessica Rees, Alexandra Burton, Kate Walters and Claudia Cooper in Dementia
