# Supplementary material for: Exploring the provision and support of care for long-term conditions in dementia: A qualitative study combining interviews and document analysis
Source: Dementia (London). 2023 Mar 7;22(4):820–37. doi: 10.1177/14713012231161854 (PMC9996169; doi:10.1177/14713012231161854)
Supplement: Supplemental Material - Exploring the provision and support of care for long-term conditions in dementia: A qualitative study combining interviews and document analysis [file sj-pdf-1-dem-10.1177_14713012231161854.pdf]

Appendix 1. Topic guide for people with dementia
Introduction

Thank you for agreeing to talk with me. We want to understand how physical health care is planned and delivered for people with dementia. We want to know what advice people involved in care provide and how people follow advice once they are at home.

To make sure that I don't miss anything, I will record our conversation on a digital recorder and then it will be transcribed. When I write it up, I will ensure that everything will be anonymous so you can't be identified.

SECTION 1: Management of/support for long-term conditions

- I'd like to start by asking you about what physical health problems you have? [prompt: diabetes, heart, lung problems?]
  - How long have you lived with [condition]?
- How do your manage your [condition]?

PROMPT: medication, insulin, inhaler, diet/fluids.

- What makes it harder to manage your [condition]?
  - PROMPT: memory, social factors, mood
  - What strategies do you use to overcome these problems?
- Do you get any help from anyone else (paid carer/family member/nurse/doctor)?
  - What do they do? PROMPT: advice, practical help, reminding, appointments
  - How often do you see/speak to them?
  - How do you keep in touch?
  - If no support, what would be helpful?

SECTION 2: Knowledge of care plan

- How do you get information/advice about what to do? Can you tell me about:
  - What advice has helped you? What it was and how it helped.
  - Any advice you haven't understood/ hasn't helped?
  - PROMPT: GP visit- What was discussed? Who attended? How was info recorded? How would others involved in care be updated?
- How would you tell your GP if your memory impacted how you manage your [condition]?
  - How could the GP change your care to make it easier to manage your [condition] due to your memory problems?
  - PROMPT: Example of changed physical health plan to account for dementia

SECTION 3: COVID-19

How has COVID-19 impacted how you manage your health? Prompt for:

- What has the pandemic made easier/harder when managing your health?
- Contact with healthcare providers/homecare workers/family members?
- Access to medical supplies? E.g. pharmacist.
- Experience of shielding? Practical- unable to get out, Psychological- worry about COVID-19.
- Experience of being cared for using PPE?

Thank you

Is there anything else you would like to add?
